# The Equilibria in Lipid–Lipoic Acid Systems: Monolayers, Microelectrophoretic and Interfacial Tension Studies

**DOI:** 10.3390/molecules25163678

**Published:** 2020-08-12

**Authors:** Paulina Laszuk, Wiesław Urbaniak, Aneta D. Petelska

**Affiliations:** 1 Faculty of Chemistry, University of Bialystok, Ciolkowskiego 1K, 15-245 Bialystok, Poland; p.laszuk@uwb.edu.pl; 2 Faculty of Mechatronics, Kazimierz Wielki University, Chodkiewicza 30, 85-867 Bydgoszcz, Poland; wurban@ukw.edu.pl

**Keywords:** lipid, lipoic acid, monolayer, liposomes, spherical bilayer, Langmuir trough, interfacial tension, microelectrophoresis, complex formation equilibria

## Abstract

In this examination, we investigated the effect of lipoic acid (LA) on the properties of biological membrane models (monolayers, bilayers, and liposomes) formed from phosphatidylcholine (PC) or phosphatidylserine (PS) using the Langmuir, microelectrophoresis, and interfacial tension methods. The Langmuir technique allowed us to calculate the π–A isotherms and determine the molecular surface areas of pure and mixed monolayers. Using mathematical equations, we established that LA and the lipids formed complexes at a 1:1 ratio. The interfacial tension method was based on Young and Laplace’s equation. We assumed the formation of a 1:1 complex in the PC–LA system. Using the mathematical relationships, we derived the parameters characterizing the resulting complex, i.e., the surface occupied by the complex and the interfacial tension and stability constant of the formed complex. The microelectrophoretic method was used to determine the dependence of the zeta potential of the lipid membranes as a function of the pH (pH 2 to 10) of the electrolyte solution. The results indicate that modification of PC or PS membranes with LA affects changes in the zeta potential and the isoelectric point values.

## 1. Introduction

Biological membranes determine the existence of every living cell. They protect their interior contents against the influence of external factors while defining their shape. Due to their complex properties and seemingly simple construction, these membranes are a mystery to many scientists. Studies on the specificity of biomembranes have been conducted since the 19th century. Despite the development of this branch of science, work with natural cell membranes continues to create problems, so research involving model systems is now being carried out. Monolayers and lipid bilayers (e.g., spherical bilayers and liposomes) are used as substitute models [1,2]. Changes in the understanding of membrane structure have occurred thanks to the use of several modern physical research techniques, including labeling atoms, electron microscopy, electron paramagnetic resonance and nuclear magnetic resonance spectroscopy, fluorescent probes, and X-ray imaging [3,4,5,6,7,8,9,10]. As mentioned above, studies are conducted using simple models to better understand the properties of natural membranes. These artificially prepared phospholipid membranes, called model membranes, are selected to mirror specific properties of biological membranes. Their composition and properties are, nonetheless, similar to those of the membranes of living cells. The types of model membranes most often used are monolayers, planar and spherical bilayers, and vesicles (liposomes).

Monolayers gained recognition as models of cell membranes only in 1917 when Irving Langmuir presented a thin surface created at the water/air interface. This created structure has become an excellent material for determining the many physicochemical parameters characterizing membranes. The properties of monolayers are studied based on measurements of their surface pressures and the surface occupied by a single molecule in a monolayer (p-A isotherm) [11,12]. 

The second types of natural membrane models are spherical lipid bilayer models. These membranes are formed by the Mueller–Rudin method [13,14]. Numerous functions of biological membranes have been reproduced and explained using these models. Many studies have demonstrated that the properties of artificially derived lipid membranes, such as interfacial tension, are very similar to those of natural cell membranes [13,14].

Liposomes, because of their construction, have been used in medicine, cosmetics, and many other branches of science. They are non-toxic, biodegradable, and have biocompatibility with biological membranes [15,16].

**Figure 1:**
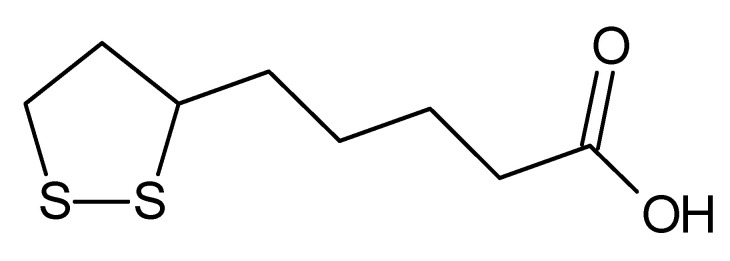
Structure of lipoic acid (LA).

Lipoic acid (LA, Figure 1) is a virtually unknown nutrient [17,18]. Lipoic acid is a sulfur-containing chemical produced in plants and animals that acts as a naturally occurring thiol antioxidant. It acts as an antioxidant both in vitro and in vivo [19,20]. LA is very useful for treating several conditions for which oxidative injury is thought to be very important, including ischemia-reperfusion, diabetes [21], cataract formation, neurodegeneration, and radiation injury [22]. Acid has also been used in the treatment of depression and Alzheimer’s disease [23]. LA can bind to Mn_2_^+^, Cu_2_^+^, Zn_2_^+^, and Pb_2_^+^ [24,25] and prevent the effects of poisoning with mercury, arsenic, cadmium, lead, and other heavy metals [26]. LA and its reduced form (DHLA) can also regenerate other endogenous antioxidants (e.g., vitamins C and E) [27].

The influence of lipoic acid on the monolayers, liposomes, and spherical bilayers formed from phosphatidylcholine (PC) or phosphatidylserine (PS) was examined in the present work. For monolayers, the interactions between the LA–PC and LA–PS systems were investigated. However, in the bilayer membrane, only the LA–PC system was examined. In these systems, the formation of the complex at a ratio of 1:1 was assumed. On this basis, the stability constants and specific surface areas of the formed complexes were determined. Furthermore, the Gibbs free energy values were calculated. Using the microelectrophoretic method, collective charts of the dependence of the zeta potential of lipid membranes as a function of pH for pure PC and PS and their mixtures were prepared with lipoic acid at different ratios. Scientists have been attempting to find, for many years, cures for several still-incurable diseases. Information about the interactions between lipoic acid and the components of natural biological membranes may contribute to the development of medicine and provide a better understanding of biological membranes’ processes. 

Today, studies have intensively focused on the interactions between membrane lipids and biologically active ingredients. This is due to the usefulness of these studies for understanding the phenomena occurring in the membranes. However, there is still a lack of a quantitative descriptions of this object. This information will be necessary for a better understanding of the processes through which place cell membranes form artificial membranes that closely resemble the properties of a biological cell. For this, it is necessary to know the molecular structure and organization of phospholipids. The data presented in this work, taken from mathematical derivations and confirmed experimentally, are of great importance for the interpretation of phenomena occurring in lipid monolayers and bilayers. These results can help us better understand the physicochemical and electric properties of biological membranes, such as the effect of bioactive components on the membrane’s surface or its zeta potential and interfacial tension. The simple and interesting methods proposed in this study can be used successfully to determine the equilibrium constant value of any 1:1 lipid–active component complex.

## 2. Results and Discussion

Lipoic acid (LA) is present in all prokaryotic and eukaryotic cells and has proven to be a potent free radical scavenger and metal chelator. LA is easily absorbed from the gastrointestinal tract, is able to cross the blood–brain barrier, and does not exhibit any serious side effects.

Understanding the influence of LA on the physicochemical properties of biological membranes is extremely important. Many experiments over the past few years have confirmed the strong potential of lipoic acid in the treatment of diabetic neuropathy, fungi and metal poisoning, and liver disorders. Nevertheless, in the literature, there are few data on the effects of lipoic acid on the physicochemical and electrical properties of model systems. The lack of quantitative descriptions of the equilibria is particularly notable.

### 2.1. Monolayer Experiment and Theoretical Considerations

In two-component monolayers (lipid (L)–lipoic acid (LA)), both substances tend to form complexes at a 1:1 ratio. This is dictated by the aspiration of the system to achieve equilibrium and thus to obtain the largest value of the stability constant [28].

The formation of a complex in a 1:1 ratio can be represented by the following equation:

(1) L+LA ⇔L–LA

The equilibrium state of the complex (1:1) is described by the following system of equations:

(2) $$a_{L}S_{L}+a_{\mathrm{LA}}S_{\mathrm{LA}}+a_{L–\mathrm{LA}}S_{L–\mathrm{LA}}=1$$

(3) $$a_{L}+a_{L–\mathrm{LA}}=c_{L}$$

(4) $$a_{\mathrm{LA}}+a_{L–\mathrm{LA}}=c_{\mathrm{LA}}$$

(5) $$K_{L–\mathrm{LA}}a_{L}a_{\mathrm{LA}}=a_{L–\mathrm{LA}}$$

(6) $$x_{\mathrm{LA}}\left(a_{\mathrm{LA}}+a_{L}\right)=a_{\mathrm{LA}}$$

where a_L_, a_LA_, and a_L–LA_ (mol m^−2^) are the surface concentrations of components L and LA, c_L_ and c_LA_ (mol m^−2^) are the total surface concentrations of components L and LA, S_L_, S_LA_, and S_L–LA_ (m^2^ mol^−1^) are the surface areas occupied by 1 mol of components L and LA and complex L–LA, K_L–LA_ (m^2^ mol^−1^) is the stability constant of complex L–LA, and x_L_ and x_LA_ are the mole fractions of components L and LA.

Solving a system of equations ultimately yields the following equations [29]:

- The equation describing the stability constant of the complex:
  (7) $$K_{L–\mathrm{LA}}=\frac{S_{\mathrm{LA}}^{3}C_{\mathrm{LA}\left(x_{\mathrm{LA}}=1\right)}^{\prime }-2S_{L}S_{\mathrm{LA}}-S_{L}^{3}c_{L\left(x_{\mathrm{LA}}=0\right)}^{\prime }}{S_{\mathrm{LA}}-S_{L}+S_{L}^{2}c_{L\left(x_{\mathrm{LA}}=0\right)}^{\prime }+S_{\mathrm{LA}}^{2}c_{\mathrm{LA}\left(x_{\mathrm{LA}}=1\right)}^{\prime }}$$
- The equation describing the area occupied by one molecule of the complex:
  (8) $$S_{L–\mathrm{LA}}=\frac{\left(S_{L}S_{\mathrm{LA}}+c_{L(x_{\mathrm{LA}=0)}}^{\prime }c_{\mathrm{LA}\left(x_{\mathrm{LA}=1}\right)}^{\prime }S_{L}^{2}S_{\mathrm{LA}}^{2}\right)\left(S_{L}+S_{\mathrm{LA}}\right)}{S_{L}^{3}c_{L\left(x_{\mathrm{LA}=0}\right)}^{\prime }+S_{\mathrm{LA}}^{3}c_{\mathrm{LA}\left(x_{\mathrm{LA}=1}\right)}^{\prime }}$$

To determine the quantities described by Formulas (7) and (8) for the experimental data, the slopes of the tangents in the ranges, including x_LA_ → 0.00 and x_LA_ → 1.00, should have been included in the above equations.

To validate the results of the stability constant and the area occupied by a molecule of the complex, the slopes of the tangents at the points x_LA_ → 0.00 and x_LA_ → 1.00 should be determined using the following equation [29]:

(9) $$C_{L\left(x_{LA=}0\right)}^{\prime }=\frac{K_{L–\mathrm{LA}}\left(S_{L}-S_{L–\mathrm{LA}}\right)-S_{L}S_{\mathrm{LA}}}{S_{L}^{2}\left(S_{L}+K_{L–\mathrm{LA}}\right)}$$

(10) $$C_{LA\left(x_{LA}=1\right)}^{\prime }=\frac{-K_{L–\mathrm{LA}}\left(S_{\mathrm{LA}}-S_{L–\mathrm{LA}}\right)-S_{L}S_{\mathrm{LA}}}{S_{\mathrm{LA}}^{2}\left(K_{L–\mathrm{LA}}-S_{\mathrm{LA}}\right)}$$

Using these formulas enabled us to compare the slopes of the curves obtained from the experimental data and calculated using the theoretical equations.

The lipid (PC or PS)–LA complex formation energy was calculated using Equation (11):

(11) $$-logK=\frac{\Delta G^{{}^{\circ}}}{2,3RT}$$

where K (m^2^ mol^−1^) is the stability constant of the lipid–LA complex, ΔG^0^ (J mol^−1^) is the lipid–LA complex formation energy, R (J mol^−1^ K^−1^) is the gas constant, and T (K) is the temperature.

In this research, we present evidence for the formation of 1:1 PC–LA and PS–LA complexes at the air/water interface. The stability constants and surfaces occupied by the PC–LA and PS–LA complexes were calculated using Equations (7) and (8).

**Figure 2:**
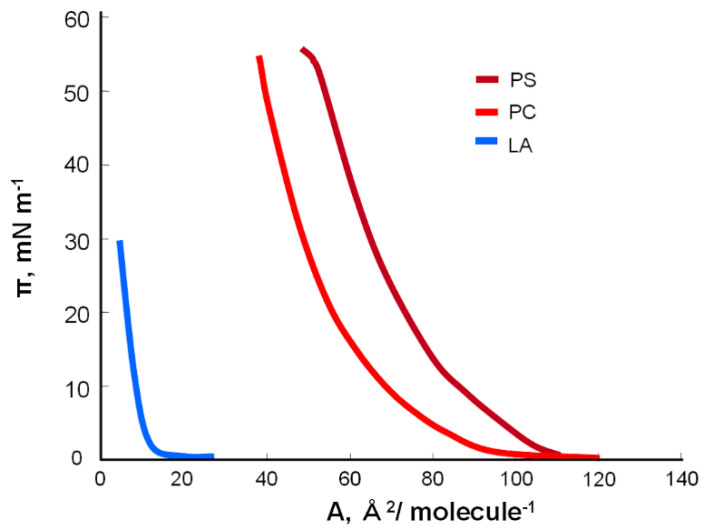
π-A isotherms of phosphatidylcholine (PC), phosphatidylserine (PS), and lipoic acid (LA).

To examine the effect of lipoic acid on the basic components of cell membranes, i.e., phosphatidylcholine and phosphatidylserine, the π-A isotherms of pure substances were prepared. The dependence of surface pressure on the surface occupied by the PC, PS, and LA molecules in the monomolecular layer is shown in the graph below (Figure 2). 

To calculate the area per molecule, a tangent to the steepest part of the curve was determined, and the value at the intersection of the line with the x-axis was read. The area occupied by one molecule is obtained experimentally by extrapolating the isotherms as π = 0. This method is the standard procedure for determining this value for monolayer experiments in the present study [30]. The surface areas for each lipid molecule were determined after the Langmuir trough was wholly covered with the lipid molecules. A further reduction in the surface does not immediately lead to the collapse or destruction of the monolayer, as changes in the orientation of the vertical chains can take place. 

For phosphatidylcholine (lecithin), this value is 64 Å^2^ molecule^−1^, which is in the range of the theoretical values [2,31,32]. The formed monolayer has three basic phase states: gaseous, liquid, and crystalline. Phosphatidylcholine formed stable monolayers, and its compression isotherm revealed a phase transition in a clear liquid expanded (LE)–liquid condensed (LC) phase, as previously described [31,32]. Molecules in the gas phase are loosely packed on the surface of the water and behave like a two-dimensional (2D) gas. During the compression of the monolayer, the gas (G)–LE is transformed. In the LE phase, the molecules behave like a 2D liquid and do not move as freely as in the gas phase. As the monolayer is compressed further, there is a phase transition LE–LC at 25 mN/m. The π-A isotherm of phosphatidylserine has a similar course to the PC isotherm. The area occupied by a single molecule in a monolayer is 78 Å^2^ molecule^−1^, but the theoretical value is 68.5–70 Å^2^ molecule^−1^ [33,34]. 

Phosphatidylcholine and phosphatidylserine were modified with lipoic acid. The area occupied by a single molecule of acid in the monolayer was much smaller than that for the two previous compounds, as illustrated by the following isotherm of α-lipoic acid. This surface is 12 Å^2^ molecule^−1^.

#### 2.1.1. PC–LA Complex

**Figure 3:**
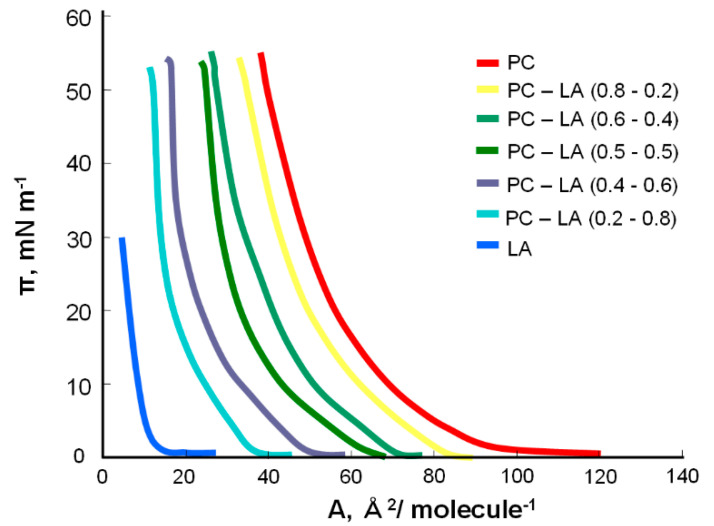
Surface pressure–area (π-A) isotherms of mixed PC–LA monolayer.

Figure 3 presents the surface pressure–area (π–A) isotherms of the mixed PC–LA monolayers with different percentages of ingredients.

The monolayers formed with different PC–LA mixtures have isotherms similar to those of the corresponding major component. These isotherms are approximately the same as each other and lie between those of pure PC and those of pure LA (Figure 3). This behavior could be caused by non-ideal mixing between the PC and LA components, likely due to the ability of the counterion to bind to LA.

It was assumed that a complex was formed between the components at a 1:1 ratio. To determine the stability constant of the K_PC–LA_ complex and the S_PC–LA_-specific surface, the relationship between the surface concentrations of phosphatidylcholine and lipoic acid was plotted as a function of the molar fraction of lipoic acid. 

**Figure 4:**
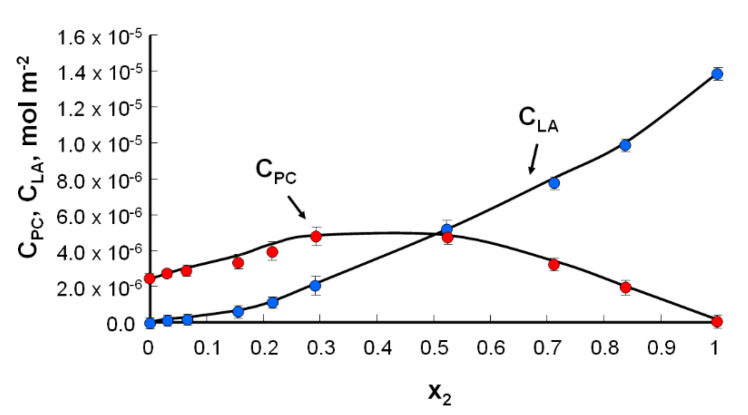
Dependence of the total surface concentration of PC (c_PC_) and LA (c_LA_) vs. the mole fraction of LA (the experimental values are indicated by the points and the theoretical values by the curve).

Using the above information, the curve of dependence of the surface concentration of phosphatidylocholine and lipoic acid vs. the molar fraction of lipoic acid was plotted (Figure 4).

Based on the equations presented above, by substituting the experimental data and using the slope of the c_PC_, c_LA_ concentration curves, the parameters characterizing the complex were determined, i.e., S_PC–LA_ = 81 Å^2^ molecule^−1^ and K_PC–LA_ = 3.5 × 10^6^ m^2^ mol^−1^. The surface of the complex formed for the phosphatidylcholine–lipoic acid system (81 Å^2^ molecule^−1^) was greater than the theoretical value (created by summing the specific surface values of pure S_PC_ + S_LA_ components = 64 Å^2^ molecule^−1^ + 12 Å^2^ molecule^−1^ = 76 Å^2^ molecule^−1^). This indicates the loose packing of the molecules in the monolayer, which may have resulted from the construction of the ingredients.

The values of the complex formation energy (Gibbs free energy) of the PC–LA complex calculated by Equation (11) were equal to −37.27 kJ mol^−1^.

#### 2.1.2. PS–LA Complex

**Figure 5:**
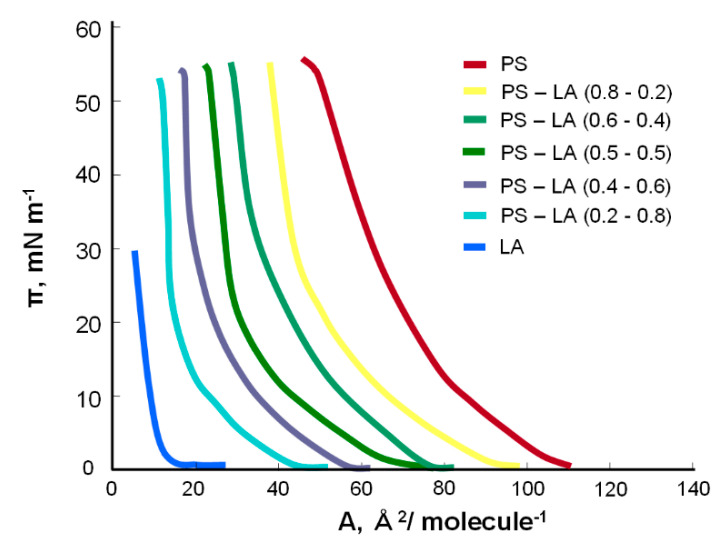
Surface pressure–area (π-A) isotherms of the mixed PS–LA monolayers.

Figure 5 presents the surface pressure–area (π–A) isotherms of the mixed PS–LA monolayers. As shown in Figure 5, the isotherm of the mixture with more PS (80:20) is similar to the pure PS but shifted to the left due to the electrostatic repulsions caused by the PS molecules present. As with the concentration of LA, the surface area occupied by one molecule decreases to that of the pure LA molecule.

**Figure 6:**
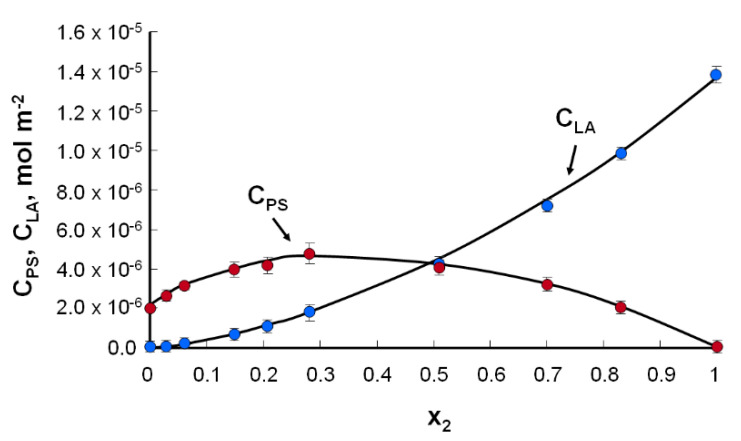
Dependence of the total surface concentration of PS (c_PS_) and LA (c_LA_) vs. the mole fraction of LA (the experimental values are indicated by points and the theoretical values by the curve).

Using the above information, the curve of dependence of the surface concentration of phosphatidylserine and lipoic acid on the molar fraction of lipoic acid was plotted (Figure 6).

To determine the stability constant of the K_PS–LA_ and S_PS–LA_ surfaces, the slopes of the curves were used in the places where the lipoic acid content was the smallest and the largest. These values were as follows: S_PS–LA_ = 86 Å^2^ molecule^−1^ and K_PS–LA_ = 8.7 × 10^6^ m^2^ mol^−1^. For the phosphatidylserine–lipoic acid system, the obtained value of the specific surface area of the complex was smaller than the theoretical result (S_PS_ + S_LA_ = 78 Å^2^ molecule^−1^ + 12 Å^2^ molecule^−1^ = 90 Å^2^ molecule^−1^), which indicates a high degree of packing and ordering among its ingredients. The high value of the stability constant of the PS–LA system indicates the creation of a constant complex.

The energy of the complex formation value for the PS–LA complex calculated from Equation (11) is equal to −39.53 kJ mol^−1^. 

The parameters characterizing the monolayers formed from the PC–LA and PS–LA complexes are presented in Table 1.

### 2.2. Microelectrophoretic Experiments

A significant parameter that controls some processes in natural cells is the zeta potential of the lipid membrane. The zeta potential depends on the molecular composition of the biological membrane and plays an essential role in understanding membrane-binding mechanisms and cellular uptake, such as drug delivery. This parameter is an essential property of a cell and is useful for comparing the surface properties between two cell types [35,36]. Changes in the zeta potential of a living cell can commonly occur, including alongside diseases in cells since the membrane zeta potential is influenced by many different factors, such as membrane composition. This contributes significantly to our understanding of many disease mechanisms because ongoing studies of natural membranes and in vitro chemical models provide valuable information about in vivo processes.

**Figure 7:**
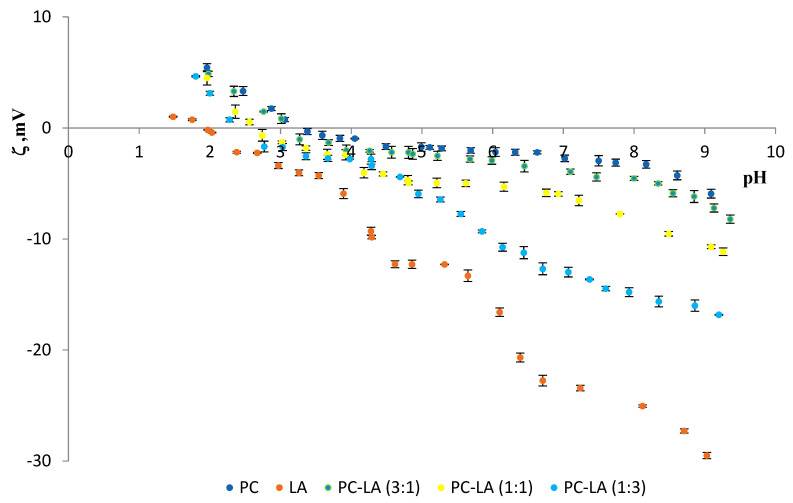
Dependence of PC and PC: LA membrane zeta potential values vs. the pH of the electrolyte solution.

**Figure 8:**
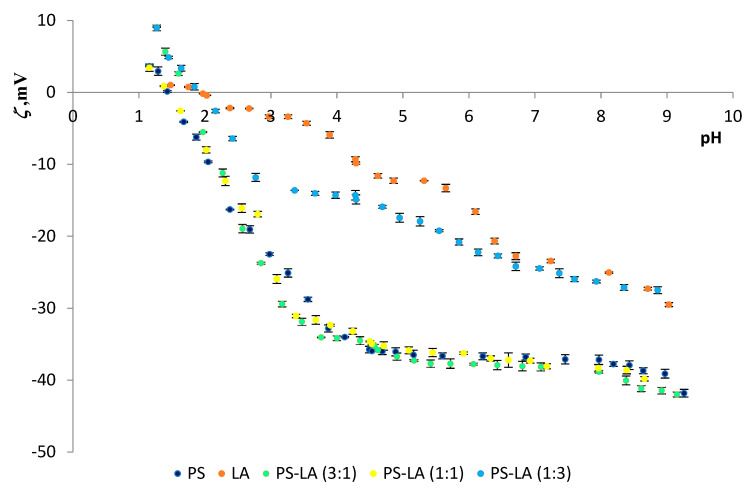
Dependence of PS and PC: LA membrane zeta potential values vs. the pH of the electrolyte solution.

The effects of lipoic acid on the zeta potential values of PC and PS membranes were also studied. The zeta potentials of the PC–LA and PS–LA mixtures are presented compared to pH (2–10) and the molar composition of the liposome components in the membranes (Figure 7 and Figure 8, respectively).

The dependence of the zeta potential on the pH of the applied electrolyte was determined for phosphatidylcholine, lipoic acid, and their mixtures at ratios of 1:3, 1:1, and 3:1 (Figure 7). It was found that with an increase in the lipoic acid content of the mixtures, the isoelectric point moved towards lower pH values. There was also a decrease in the zeta potential values at pH~2 and an increase in the negative zeta potential values at pH~9.

The system of phosphatidylserine–lipoic acid (Figure 8) was examined analogously. It was found that as the content of lipoic acid increased in the tested mixtures, the isoelectric point moved towards higher pH values. In the phosphatidylserine–lipoic acid system, at ratios of 1:1 and 3:1, the addition of acid slightly affected the course of the obtained curves in the curve obtained for pure PS. However, there was a decrease in the negative zeta potential values at pH~2 and an increase in the negative zeta potential values at pH~9. For the PS–LA system at a ratio of 1:3, there was a marked decrease in negative zeta potential values for both low and high pH values.

Table 2 and Table 3 recapitulate the results for the effect of liposome composition on the isoelectric points and the zeta potential values for the PC–LA and PS–LA systems, respectively.

Modifying PC or PS membranes with lipoic acid causes changes in their zeta potential values (Table 2 and Table 3, and Figure 7 and Figure 8). 

Comparing the two tested systems, PC–LA and PS–LA, the following conclusions can be made:

- The isoelectric point for the anionic PS lipid with lipoic acid (e.g., for 1:1 ratio) lies at lower pH values (~1.5) compared to the PC–LA system (pH ~ 2.6).
- The zeta potential values for the PS–LA system in the range of pH 2–9 are negative and show values from −3 to −40 mV, while for the PC–LA system, these values are lower and are 5 mV (for pure PC at pH~2) to −17 mV (for the PC–LA mixture (1: 3)).

The liposome zeta potential depends on the electrolyte ions and electrolyte concentrations used in the measurements, as well as the pH and membrane composition. In this paper, we demonstrated that a modification of the PC or PS membranes with lipoic acid causes changes in the analyzed parameters characterizing the model lipid membranes. Zeta potential is a standard measurement for nanoparticle surface characterization and depends on a host of parameters, including temperature, pH, conductivity (ionic strength), and solvent (viscosity). Liposomes are one of the most commonly used nanomedicine drug delivery vehicles (for example, biologically active components), and PC is the most commonly used lipid for the preparation of liposomes. The findings from this study can be used to inform the drug development and formulation processes. Despite the fact that the presented studies are preliminary, we hope that the description of lipoic acid interactions with natural membranes will contribute to this acid’s use as a therapeutically active substance. There is certainly a need for further studies to determine information that may be helpful in solving a number of problems facing biophysics, biochemistry, and medicine.

### 2.3. Interfacial Tension Experiment and Theoretical Considerations

A solution used for forming artificial spherical bilayers can contain one, two, or several components. We started our analysis with a 1:1 complex because complexes with this stoichiometry have maximal values for the stability constant. The fact that the first stability constant in the complex, as the most essential one, is usually the biggest, should also be taken into consideration. In the initial stage of the complexing reactions, the 1:1 complex is formed, and in the following stages, other complexes can be formed. In our case, the equation derived to describe the equilibria of the 1:1 complex formation was sufficient for the whole range of concentrations. 

We also considered the case in which the components of a two-component lipid membrane, e.g., PC or PS (component 1), and lipoic acid (component 2) do not form a new chemical compound. The interactions between the membrane components can then be described by a system of equations [35,37]:

(12) $$\gamma_{1}m_{1}A_{1}+\gamma_{2}m_{2}A_{2}=\gamma$$

(13) $$\frac{m_{1}}{m_{1}+m_{2}}=x_{1}$$

(14) $$x_{1}+x_{2}=1$$

where $A_{1}^{-1}$ and $A_{2}^{-1}$ (mol m^−2^) are the surface concentrations of bilayer components 1 and 2, $m_{1}$ and $m_{2}$ (mol m^−2^) are the quantities of bilayer components 1 and 2 per unit area of the membrane, $\gamma_{1}$ and $\gamma_{2}$ (N m^−1^) are the interfacial tensions of the membranes formed from pure components 1 and 2, γ (N m^−1^) is the measured interfacial tension of the bilayer membrane, and $x_{1}$ and $x_{2}$ are the solution mole fractions of bilayer components 1 and 2.

By eliminating parameters $m_{1}$ and $m_{2}$, a straight-line equation is obtained:

(15) $$\left(\gamma -\gamma_{1}\right)x_{1}=\frac{A_{2}}{A_{1}}\left(\gamma_{2}-\gamma \right)x_{2}\;$$

**Figure 9:**
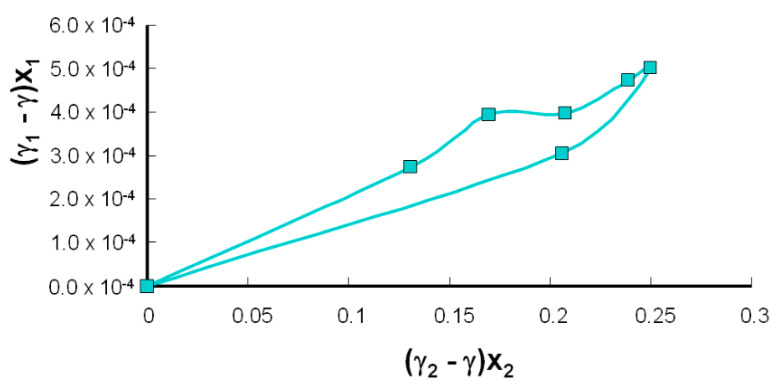
A plot of Equation (15) for PC–LA, where x_1_ and x_2_ are the mole fractions of components 1 and 2 (PC and LA, respectively), showing the interfacial tension measurements. PC: phosphatidylcholine; LA: lipoic acid.

Figure 9 provides graphs for the system (i.e., the PC–LA). When there is no interaction between lipids and lipoic acid, these functions should give straight lines (Equation (15)). Obviously, this is not the case, suggesting that there is a complex or different structure in the PC–LA system. Since Equation (15) assumes the formation of a 1:1 complex, our initial assumption was that these components form a complex between the components of the membrane. The examined system was analyzed over the entire possible range of component compositions to obtain their interfacial tension values.

The dependence of interfacial tension on membrane composition for the PC–LA membrane was analyzed using the possible concentration range. The interfacial tension of the PC–LA membrane was determined compared to the composition with 60% of the lipoic acid content because a bilayer was created only with this amount of component 2 (LA) with PC. 

The pure PC interfacial tension value (component 1), γ_1_, was determined directly [37] and is equal to 1.62 ± 0.12 × 10^−3^ Nm^−1^. Since lipoic acid (component 2) does not create a bilayer, there are no accurate interfacial tension data for this acid in the literature.

**Figure 10:**
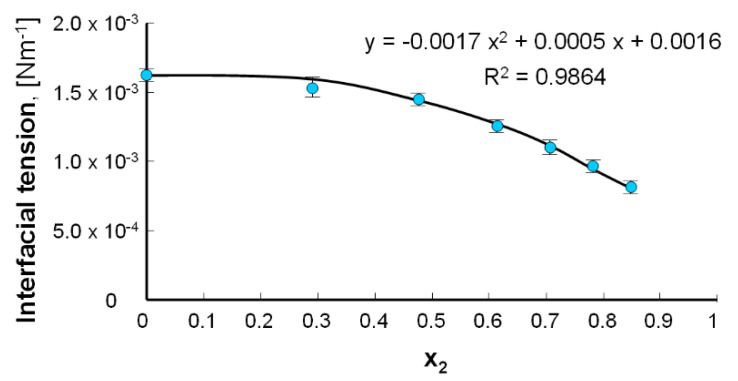
The hypothetical interfacial tension value for the lipoic acid bilayer.

To describe the course of the experimental curve, the γ_2_ value of the pure component was necessary. Therefore, the hypothetical interfacial tension data for the LA membrane was calculated by adjusting the experimental curve by the polynomial of the other mark, extrapolating the x_2_ = 1 value (Figure 10). This value is equal to 4.30 × 10^−4^ Nm^−1^. Such a low interfacial tension value confirms that it is not possible to create a pure lipoic acid bilayer. 

**Figure 11:**
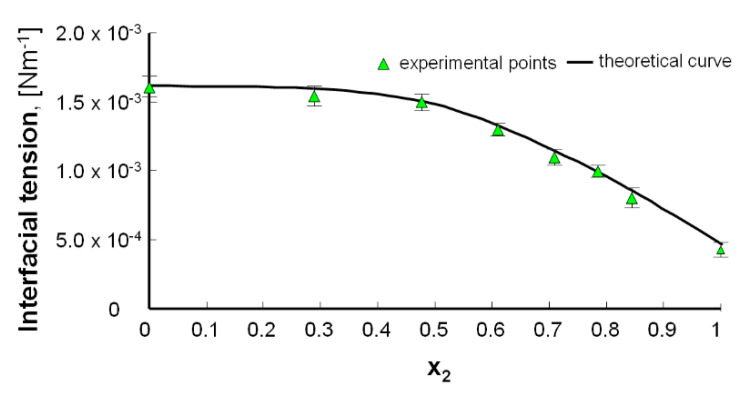
PC–lipoic acid interfacial tension data vs. the mole fraction of lipoic acid. The experimental values are marked by points, and the theoretical values are marked by curves.

Figure 11 shows the interfacial tension of a PC–LA membrane vs. the mole fraction of lipoic acid. The interfacial tension values of the PC and LA membranes are 1.62 × 10^−3^ N m^−1^ and 0.43 × 10^−3^ N m^−1^, respectively.

The curve presented in Figure 9 should be a straight line (Equation (15)), with no interactions. The nonlinear nature of the curve indicates interactions between PC and lipoic acid. 

Therefore, using previous research, we assumed that the membranes could also allow the two components to form a complex. Complexes with different stoichiometries can form between the membrane components. Since the first stability constant in these complexes is usually the largest [38], we assumed that the complexes being formed have a 1:1 composition. The interactions between the bilayer components can be described using a previously published set of equations [13] for bilayer components that form a 1:1 complex. 

The basic equation describing the interactions between the phosphatidylcholine and lipoic acid is presented below:

(16) $$\left[\left(\gamma -\gamma_{1}\right)B_{2}x_{1}+\left(\gamma -\gamma_{2}\right)B_{1}x_{2}\right]\left[\left(\gamma_{3}-\gamma_{1}\right)B_{2}x_{1}+\left(\gamma_{3}-\gamma_{2}\right)B_{1}x_{2}+\left(\gamma_{1}-\gamma_{2}\right)\left(x_{1}-x_{2}\right)\right]\;=KA_{3}^{-1}B_{1}B_{2}\left[\left(\gamma -\gamma_{1}\right)\left(x_{2}-x_{1}\right)+\left(\gamma_{3}-\gamma \right)B_{1}x_{2}\right]\left[\left(\gamma -\gamma_{2}\right)\left(x_{1}-x_{2}\right)+\left(\gamma_{3}-\gamma \right)B_{2}x_{1}\right]$$

where $B_{1}=\frac{A_{3}}{A_{1}}$ and $B_{2}=\frac{A_{3}}{A_{2}}\;$ and $A_{3}^{-1}$ (mol m^−2^) are the surface concentrations of the 1:1 complex, $\gamma_{3}$ (N m^−1^) is the interfacial tensions of the 1:1 complex, and K (m^2^ mol^−1^) is the stability constant of the surface of the 1:1 complex.

Equation (16) can be simplified by assuming that the stability constant of the formed complex has a high value. Then, one can apply simplifications that allow Equation (16) to handle the linear behavior for small (x_2_ < x_1_) and large (x_2_ > x_1_) x_2_ values:

(17) $$\left(\gamma_{1}-\gamma \right)\frac{x_{1}-x_{2}}{x_{2}}=-B_{1}\gamma_{3}+B_{1}$$

(18) $$\left(\gamma_{2}-\gamma \right)\frac{x_{2}-x_{1}}{x_{1}}=-B_{2}\gamma_{3}+B_{2}$$

To calculate the stability constant of the complex, Equation (16) can be simplified to $x_{1}=x_{2}$.

(19) $$K{\left(A_{1}^{-1}\right)}^{2}{\left(A_{2}^{-1}\right)}^{2}{\left(A_{3}^{-1}\right)}^{-1}{\left(\gamma -\gamma_{3}\right)}^{2}=\left[\gamma_{2}A_{1}^{-1}+\gamma_{1}A_{2}^{-1}-\gamma \left(A_{1}^{-1}+A_{2}^{-1}\right)\right]\;(\gamma_{2}A_{1}^{-1}+\gamma_{1}A_{2}^{-1})-\left[\gamma_{2}A_{1}^{-1}+\gamma_{1}A_{2}^{-1}-\gamma \left(A_{1}^{-1}+A_{2}^{-1}\right)\right]\left(A_{1}^{-1}+A_{2}^{-1}\right)\gamma_{3}$$

Equation (20) is used to calculate the theoretical interfacial tension values based on their agreement between the experimental and theoretical data:

(20) $$KA_{1}^{-1}A_{2}^{-1}\left(a_{1}+a_{2}\right)\left(a_{3}-a_{1}\right)\gamma^{2}+{\left[KA_{1}^{-1}A_{2}^{-1}\left(\gamma_{1}a_{1}-\gamma_{3}a_{3}\right)\left(a_{1}+a_{2}\right)-\;KA_{1}^{-1}A_{2}^{-1}\left(\gamma_{2}a_{1}+\gamma_{3}a_{2}\right)\left(a_{3}-a_{1}\right)+a_{4}A_{3}^{-1}\left(a_{3}+a_{2}\right)\right]}^{}\gamma ++KA_{1}^{-1}A_{2}^{-1}a_{3}\gamma_{3}\left(\gamma_{3}a_{2}+\;\gamma_{1}a_{2}\right)-KA_{1}^{-1}A_{2}^{-1}a_{1}\gamma_{1}\left(a_{1}\gamma_{2}+a_{2}\gamma_{3}\right)-a_{4}A_{3}^{-1}\left(\gamma_{2}a_{3}+\gamma_{1}a_{2}\right)=0$$

where:

$a_{1}=A_{3}^{-1}\left(x_{2}-x_{1}\right)$

$a_{2}=A_{2}^{-1}x_{1}$

$a_{3}=A_{1}^{-1}x_{2}$

$a_{4}=\left[A_{3}^{-1}\left(\gamma_{1}-\gamma_{2}\right)\left(x_{2}-x_{1}\right)+\left(\gamma_{1}-\gamma_{3}\right)x_{1}A_{2}^{-1}+\left(\gamma_{2}-\gamma_{3}\right)x_{2}A_{1}^{-1}\right]$

The creation of a 1:1 complex helps explain the deviation from the additivity rule in the case of two-component systems. Theoretical curves were obtained using parameters characterizing the complexes, such as the stability constants, molecular areas, and interfacial tension values. The accuracy of the presented models was verified by comparison with the experimental data.

**Figure 12:**
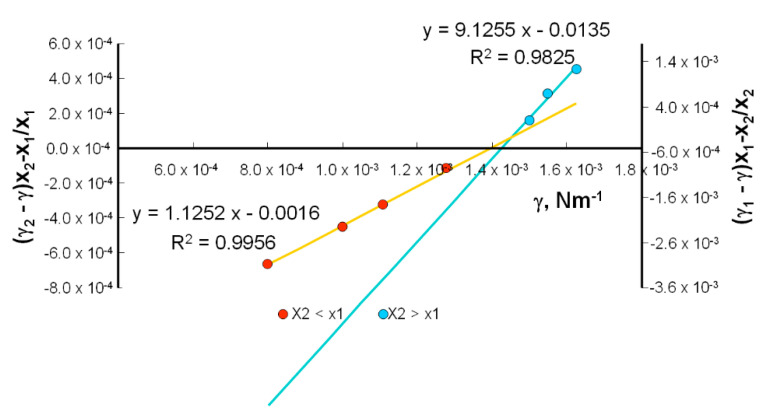
A plot showing Equations (17) and (18) for determining B_1_, B_2_, and γ: parameters of the PC–LA system.

Plots of Equations (17) and (18) are presented in Figure 12. From the slopes of the lines, the relevant values were obtained. The intersections of the straight lines with the ordinate were used to determine γ_3_, the interfacial tension of the PC–LA complex. The obtained mean value was 1.43 × 10^−3^ Nm^−1^. 

Determining the interfacial tension as a function of composition enabled us to calculate the surface concentrations for the bilayers formed from PC and LA. At least one of these calculations is necessary to determine the value of $A_{3}^{-1}$. The surface areas occupied by PC and LA are 85.0 ± 0.9 Å^2^ molecule^−1^ [38] and 12 ± 0.1 Å^2^ molecule^−1^ (obtained by the Langmuir method), respectively. The surface concentrations of PC and lipoic acid in the bilayers are 1.95 × 10^−6^ molm^−2^ and 1.38 × 10^−5^ molm^−2^, respectively. Knowledge of $A_{1}^{-1}$ and $A_{2}^{-1}$ as well as $B_{1}$ and $B_{2}$ is needed to determine the surface concentration of a membrane composed of a PC–LA complex. The obtained value, $A_{3}^{-1}$, for the PC–LA complex was 1.69 × 10^−6^ molm^−2^. Based on this value, the area occupied by one PC–LA complex is approximately 98 Å^2^ molecule^−1^. This value is almost the same as the sum of the areas occupied by individual PC and lipoic acid molecules. 

Using Equation (19), the stability constant of the PC–lipoic acid complex was obtained by setting $x_{1}=x_{2}=0.5$, and the value was 9.20 × 10^6^ m^2^ mol^−1^. To our knowledge, no stability constant for the PC–LA complex has been reported to date.

Based on the fact that the obtained value is relatively high, we can conclude that the 1:1 complex was formed in the mixed PC–lipoic acid bilayer. The K value obtained for the PC–LA monolayers (3.5 × 10^6^ m^2^mol^−1^) was about one order of magnitude smaller than the K values for the bilayers. The difference between these values is relatively large if we consider their different spatial surroundings.

As calculated by Equation (11), the complex formation energy (Gibbs free energy) values for the PC–LA complex was equal to −39.74 ± 1.05 kJ mol^−1^. Table 4 presents the calculated parameter values for the PC–LA complex. 

The theoretical data (obtained from Equation (20)), denoted by lines, and the experimental data, denoted by points, are illustrated in Figure 11. Considering the good agreement between the theoretical and experimental values, it can be concluded that a 1:1 PC–LA complex was formed in the bilayer.

During our investigations, we assumed the formation of a PC–lipoic acid complex in both the monolayer and bilayer. These complexes arise by producing a connection between the $-\overset{\left(+\right)}{N}{\left(CH_{3}\right)}_{3}$ group from the molecule of the phosphatidylcholine and $-COO^{\left(-\right)}$ groups of lipoic acid. The dissociation constants of the $-\overset{\left(+\right)}{N}{\left(CH_{3}\right)}_{3}$ group from the PC and $-COO^{\left(-\right)}$ groups of the lipoic acid are equal to 10^−5.7^ [38] and about 10^−5^ [39], respectively. 

The experimentally obtained value for the area occupied by the PC–lipoic acid complex for the mixed monolayer is 81.0 ± 0.8 Å^2^ molecule^−1^, and the value obtained for the area occupied by the PC–lipoic acid complex in the bilayer is 98.0 ± 0.9 Å^2^ molecule^−1^. This difference is likely connected to the arrangement of PC molecules in this complex and is connected to the structural construction of such complexes in the bilayers. In Reference [40], we suggested an arrangement of lecithin molecules in the bilayer membrane at pH > 5. In these media, one particle from the lecithin molecules in the bilayer (orientated in this way) has two straightened chains; however, the next molecule of lecithin has one straightened and another chain fastened to the membrane surface. An association of ions occurs under such conditions with OH^−^ from the electrolyte solution. These ions were previously characterized in Reference [40]. These ions are strongly solvated and produce a separation of lecithin particles in the bilayer, which has an influence on increasing the surface occupied by a single molecule of lecithin.

The stability constant of the PC–LA complex (monolayer) is 3.5 × 10^6^ m^2^ mol^−1^, whereas the stability constant of the PC–LA complex (for the bilayer) is 9.2 × 10^6^ m^2^ mol^−1^. These values are relatively high, which further confirms the validity of the hypothesis on the formation of 1:1 complexes, both in monolayers and in two-component layers. The difference between these values is quite large if we consider their different spatial surroundings. The higher value of the complex stability constant for the bilayer (about two-times higher) is due to its additional strengthening via interactions between the two layers of the bilayer [41].

Knowledge of this thermodynamic function provides information concerning the nature and type of bonding in the tested systems and groups taking part in the complex-forming reactions, for which there are many areas of application in chemistry, biology, and medicine. The complex formation energy values for PC–LA in the monolayer and bilayer systems are −37.27 ± 1.64 and −39.68 ± 1.75 kJ mol^−1^, respectively. The values presented above are close to those determined earlier for similar systems (e.g., PC–decanoic acid or stearic acid) [42,43].

## 3. Materials and Methods 

### 3.1. Materials 

Membrane-forming materials: Phosphatidylcholine from soybean (≥97%, Sigma-Aldrich, St. Louis, MO, USA), prepared according to a modification of the procedure of Singleton et al. [44], was used in the experiment. Phosphatidylserine (≥97%) and lipoic acid (99%) were purchased from Sigma-Aldrich (St. Louis, MO, USA) and were used as received. The molecular weights of the PC, PS, and LA were approximately 752.08, 792.07, and 206.33 g mol^−1^, respectively.

Electrolyte solutions: The electrolyte solutions for monolayer (pure water), interfacial tension measurements (0.1 M KCl), and microelectrophoresis (0.155 M NaCl) were prepared using water purified through a Milli-Q plus water purification system (Millipore, Burlington, MA, USA) with a resistivity of 18.2 MΩ cm.

#### 3.1.1. Monolayer Preparation—Spreading Solvent and Subphase 

The 1-chloropropane solvent (>98%) was supplied by Sigma-Aldrich (St. Louis, MO, USA) and used as a spreading solvent for the substances. The spreading solution contained the appropriate amount of each material in 1-chloropropane (1 mg cm^−3^ concentration). An ultrapure water subphase solution was used as the subphase.

#### 3.1.2. Spherical Bilayer Preparation

Spherical bilayer-forming solutions: PC and LA were purified by dissolution in chloroform (anhydrous, ≥99%); then, the solvent was evaporated under argon. The stock solutions for spherical bilayer formation were composed of 20 mg·cm^−3^ of the components (PC, LA) in n-decane and butanol (anhydrous, ≥99%) at a ratio of 10:1. The stock solution containing the components was stored at 4 °C.

#### 3.1.3. Liposome Preparation

Liposome-forming solutions: The solutions for liposome formation were composed of 10 mg·cm^−3^ of the substances (PC, PS, LA) in n-chloroform (anhydrous, ≥99%). The components were mixed in three molar ratios (3:1, 1:1, and 1:3). Then, the n-chloroform was evaporated under a stream of argon to obtain a dry residue. The resulting residue was hydrated with an electrolyte solution (0.155 M NaCl).

Preparation of liposomes: Liposomes were prepared by sonicating the suspension. For liposome formation, solutions (concentration 10 mg cm^−3^) of phosphatidylcholine, phosphatidylserine, lipoic acid, and their mixtures at a certain weight ratio were used alongside argon to evaporate the solvent and protect the resulting layer from oxidation. Next, we added 5 mL 0.9% NaCl, and, using an ice bath, sonication was carried out (5 times for 1.5 min) with a UD-20 ultrasonic disintegrator (Techpan, Pulawy, Poland).

### 3.2. Monolayer Measurements

The working conditions and experimental procedure: The surface tension was measured at the air/water interface (22 °C) using a homemade computer-controlled apparatus, as described in the previous papers [29,31]. 

The monolayers were prepared using a micro-syringe to spread a calculated volume of the substance in 1-chloropropane on the aqueous subphase. A total of 15 min was allowed to evaporate the spreading solvent and monolayer equilibration before starting the measurements.

Then, the monolayer was subjected to continuous compression with a glass barrier to obtain π-A isotherms. However, glass is a hydrophilic material that does not allow phospholipids to pass under its barrier.

The surface tension results were expressed as the surface pressure vs. area per molecule (π–A) isotherms. These measurements were carried out using the Langmuir method. The apparatus consisted of a Nima tensiometer, a Teflon trough with a surface area of 648 cm^2^, a glass barrier, a thin plate, a moving barrier system, and the control unit of the tensiometer. The barrier was set to move at a velocity of 0.04 cm s^−1^. A computer program (Nima ST9002) was used to calculate the surface pressure of the monolayer (π) as a function of the surface area per molecule (A): π = γ_0_ − γ = f(A), where γ_0_ is the surface tension of the bare air/water interface, and γ is the surface tension of the lipid-covered surface. Before the experiment was started, the Teflon trough was carefully cleaned and rinsed with ultrapure water. The experimental system was enclosed in an acrylic box to minimize water evaporation, ensure high humidity, and avoid contamination. The reported values were highly reproducible and represented the average of at least five experiments. Standard deviations for the measurements were less than 1%.

### 3.3. Interfacial Tension Measurements

Working conditions and experimental procedure: The interfacial tension measurements were based on Young’s and Laplace’s equation presented in the previous paper [35]. 

The apparatus and method of measurement were both described in detail previously [35]. The measurement cell was built from two glass elements separated by a mount holding (1.5 mm diameter) a circular Teflon cap that was axially pierced, leaving a small orifice. The Mueller–Rudin method [35,37] was used to form the bilayers at the flat end of the Teflon cap. Both glass elements were filled with 0.1 M KCl. The stock solution was introduced into the flat wall of the Teflon cap, and pressure was applied to the left glass element of the measuring cell using a manometer.

The convexity of the bilayer cap was measured to a 0.05 mm precision by the optical part of the measuring apparatus [35]. Over-pressure causing spherical bilayer convexity was determined using the manometer. The interfacial tension was measured on a bilayer 12–15 times. Measurements with the preparation of the electrolyte solution were made 2–3 times to test the repeatability. The experimental results are presented with error bars in Figure 10 and Figure 11. All presented experiments were carried out at a temperature of 293 ± 2 K.

### 3.4. Microelectrophoretic Measurements

The reason for using the microelectrophoresis method is to obtain a graph of the zeta potential’s dependence on the pH value. The use of the Zetasizer Nano ZS (Malvern Instruments, Malvern, United Kingdom) apparatus allowed us to obtain data on the zeta potential and microelectrophoretic mobility of the studied systems using the laser Doppler Micro-electrophoresis (LDE) technique. All measurements were performed as a function of pH using a WTW InoLab pH 720 laboratory meter (WTW, Weinheim, Germany). Liposomes suspended in the electrolyte solution (0.155 mol dm^−3^ NaCl) were titrated to the given pH value (range 2–10, every ± 0.3 units) with sodium hydroxide or hydrochloric acid. Six measurements were made (each covering 100–200 series for a duration of 5 s) for each pH value for each sample. The experiments were carried out three times [16,35,36]. 

## 4. Conclusions

The presented data relate to the physicochemical and electrical properties of phospholipids modified with lipoic acid, indicating interactions between the components of the surrounding solution and between the membrane components. The interfacial tension of the lipid bilayer measurements demonstrates the good compliance between the theoretical and experimental data (the assumption of a 1:1 complex). Similar relationships were obtained for the monolayers. The good agreement between the experimental points and theoretical ones indicates that the theoretical models proposed by us (Section 2.1 and Section 2.3) can sufficiently describe the interactions in PC–lipoic acid (monolayer and spherical bilayers) and PS–lipoic acid (monolayers) membranes. The mathematically calculated and experimentally confirmed data presented in this work are important for the understanding of various phenomena in both monolayers and lipid bilayers. The zeta potential of PC or PS liposomes depends on the modification of their structures, found here by examining the lipoic acid and pH of the electrolyte solution. A shift of the isoelectric point was also observed. We believe that the solution to even a seemingly small problem associated with cell membranes can help solve other unexplained puzzles in the human body. The results presented in this paper will be important to learn about the properties of cell membranes and the mechanisms involved in their participation.

In conclusion, the value of the stability constants of the lipid–lipoic acid complexes in model lipid membranes (monolayer, spherical bilayer, and liposomes) was reported for the first time in this paper.

## Figures and Tables

**Table 1:** Calculated physicochemical parameters for examined complexes: phosphatidylcholine–lipoic acid (PC–LA) and phosphatidylserine–lipoic acid (PS–LA).

System	Surface Area of Complex (Ǻ^2^ molecule^−1^)	Stability Constant (m^2^·mol^−1^)	Complex Formation Energy (kJ mol^−1^)
**PC–LA**	81 ± 0.8	3.50 × 10^6^	−37.27 ± 1.64
**PS–LA**	86 ± 0.9	8.70 × 10^6^	−39.53 ± 1.74

**Table 2:** The zeta potential values and isoelectric points for the PC-LA system.

Examined System	Isoelectric Point	Zeta Potential Values ς (mV)	
		pH~2	pH~9
PC	~3.2	5.47 ± 0.33	−5.93 ± 0.40
PC–LA (3:1)	~3.1	2.03 ± 0.27	−7.20 ± 0.27
PC–LA (1:1)	~2.6	4.53 ± 0.60	−10.73 ± 0.20
PC–LA (1:3)	~2.3	3.13 ± 0.20	−16.80 ± 0.33

**Table 3:** The zeta potential values and isoelectric points for the PS–LA system.

System	Isoelectric Point	Zeta Potential Values ς (mV)	
		pH~2	pH~9
PS	~1.4	−9.66 ± 0.13	−39.13 ± 0.60
PS–LA (3:1)	~1.7	−5.53 ± 0.07	−42.04 ± 0.35
PS–LA (1:1)	~1.5	−8.02 ± 0.40	−40.35 ± 0.59
PS–LA (1:3)	~1.9	−2.60 ± 0.35	−28.35 ± 0.36

**Table 4:** Some physicochemical data for the PC–LA complex.

System	Surface Area (Ǻ^2^ Molecule^−1^)	Stability Constant (m^2^ mol^−1^)	Complex Formation Energy (kJ mol^−1^)
PC–LA	98.0 ± 0.9	9.20 × 10^6^	−39.68 ± 1.75

## References

[1] Singer S.J., Nicolson G.L. (1972). The Fluid Mosaic Model of the Structure of Cell Membranes. Science.

[2] Tien H.T., Ottova-Leitmannova A. (2003). Planar Lipid Bilayers (BLM’s) and Their Applications.

[3] Kleusch C., Hersch N., Hoffmann B., Merkel R., Csiszár A. (2012). Fluorescent Lipids: Functional Parts of Fusogenic Liposomes and Tools for Cell Membrane Labeling and Visualization. Molecules.

[4] Siontorou C.G., Nikoleli G.-P., Nikolelis D.P., Karapetis S.K. (2017). Artificial Lipid Membranes: Past, Present, and Future. Membranes.

[5] Schrank E., Wagner G.E., Zangger K. (2013). Solution NMR Studies on the Orientation of Membrane-Bound Peptides and Proteins by Paramagnetic Probes. Molecules.

[6] Blake H.L., Robinson D. (2014). QM/MM Studies of Contemporary and Novel Membrane Raft Fluorescent Probes. Molecules.

[7] Kučerka N., Heberle F.A., Pan J., Katsaras J. (2015). Structural Significance of Lipid Diversity as Studied by Small Angle Neutron and X-ray Scattering. Membranes.

[8] Mottola M., Caruso B., Perillo M.A. (2019). Langmuir films at the oil/water interface revisited. Sci. Rep..

[9] Dabkowska A.P., Valldeperas M., Hirst C., Montis C., Pálsson G.K., Wang M., Nöjd S., Gentile L., Barauskas J., Steinke N.-J. (2017). Non-lamellar lipid assembly at interfaces: Controlling layer structure by responsive nanogel particles. Interface Focus..

[10] Clara Botarelli Kabbach C.B., Gonçalves dos Santos R. (2018). Effects of pH and Temperature on the Phase Behavior and Properties of Asphaltene Liquid Films. Energy Fuels.

[11] Wnętrzak A., Łątka K., Makyła-Juzak K., Zemla J., Dynarowicz-Łątka P. (2015). The influence of an antitumor lipid – erucylphosphocholine – on artificial lipid raft system modeled as Langmuir monolayer. Mol. Membr. Biol..

[12] Wojciechowski K., Orczyk M., Gutberlet T., Brezesinski G., Geue T., Fontaine P. (2016). On the Interaction between Digitonin and Cholesterol in Langmuir Monolayers. Langmuir.

[13] Petelska A.D. (2012). Interfacial tension of bilayer lipid membrane. Cent. Eur. J. Chem..

[14] Petelska A.D., Naumowicz M., Figaszewski Z.A. (2011). The interfacial tension of the lipid membrane formed from lipid-amino acid systems. Cell Biochem. Biophys..

[15] Stebelska K., Wyrozumska P., Grzybek M., Sikorski A.F. (2002). Characterization and medical applications of liposome constructions. Adv. Clin. Exp. Med..

[16] Kotyńska J., Figaszewski Z.A. (2007). Adsorption equilibria at interface separating electrolyte solution and phosphatidylcholine-stearylamine liposome membrane. Biophys. Chem..

[17] Turkowicz M., Jastrzebska I., Hryniewicka M., Kotowska U., Gudalewska D., Karpińska J. (2020). Investigation of Lipoic Acid - 4-methoxybenzyl Alcohol Reaction and Evaluation of Its Analytical Usefulness. Food Chem..

[18] Wołyniec E., Karpińska J., Losiewska S., Turkowicz M., Klimczuk J., Kojło A. (2012). Determination of lipoic acid by flow-injection and high-performance liquid chromatography with chemiluminescence detection. Talanta.

[19] McNeilly A.M., Davison G.W., Murphy M.H., Nadeem N., Trinick T., Duly E., Novials A., McEneny J. (2011). Effect of α-lipoic acid and exercise training on cardiovascular disease risk in obesity with impaired glucose tolerance. Lipids Health Dis..

[20] Rochette L., Ghibu S., Richard C., Zeller M., Cottin Y., Vergely C. (2013). Direct and indirect antioxidant properties of α-lipoic acid and therapeutic potential. Mol. Nutr. Food Res..

[21] Jacob S., Henriksen E.J., Schiemann A.L., Simon I., Clancy D.E., Tritschler H.J., Jung W.I., Augustin H.J., Dietze G.J. (1995). Enhancement of glucose disposal in patients with type 2 diabetes by alpha-lipoic acid. Arzneimittelforschung.

[22] Packer L., Witt E.H., Tritschler H.J. (1995). alpha-Lipoic Acid as a Biological Antioxidant. Free Radic. Biol. Med..

[23] Galasko D.R., Peskind E., Clark C.M., Quinn J.F., Ringman J.M., Jicha G.A., Cotman C., Cottrell B., Montine T.J., Thomas R.G. (2012). Antioxidants for Alzheimer disease: A randomized clinical trial with cerebrospinal fluid biomarker measures. Arch. Neurol..

[24] Sigel H., Prijs B., McCormick D.B., Shih J.C. (1978). Stability and structure of binary and ternary complexes of alpha-lipoate and lipoate derivatives with Mn^2+^, Cu^2+^, and Zn^2+^ in solution. Arch. Biochem. Biophys..

[25] Ou P., Tritschler H.J., Wolff S.P. (1995). Thioctic (lipoic) acid: A therapeutic metal-chelating antioxidant?. Biochem. Pharmacol..

[26] Patrick L. (2003). Toxic metals and antioxidants: Part II. The role of antioxidants in arsenic and cadmium toxicity. Altern. Med. Rev..

[27] Shay K.P., Moreau R.F., Smith E.J., Smith A.R., Hagen T.M. (2009). Alpha-lipoic acid as a dietary supplement: Molecular mechanisms and therapeutic potential. Biochim. Biophys. Acta.

[28] Inczedy J. (1976). Analytical Applications of Complex Equilibria.

[29] Janicka K., Jastrzębska I., Petelska A.D. (2016). The equilibria of diosgenin-phosphatidyolcholine and diosgenin-cholesterol in monolayers at the air/water interface. J. Membrane Biol..

[30] Birdi K.S. (1989). Lipid, and Biopolymer Monolayers at Liquid Interfaces.

[31] Janicka K., Szeremeta M., Petelska A.D. (2019). Equilibrium of phosphatidylcholine-ergosterol in monolayers at the air-water interface. J. Chem. Thermodyn..

[32] Sovago M., Wurpel G.W.H., Smits M., Müller M., Bonn M. (2007). Calcium-induced phospholipid ordering depends on surface pressure. J. Am. Chem. Soc..

[33] Petelska A.D., Figaszewski Z.A. (2003). Acid-base equilibria at interface separating electrolyte solution and lipid bilayer formed from phosphatidylserine. Biophys. Chem..

[34] Luna C., Stroka K.M., Bermudez H., Aranda-Espinoza H. (2011). Thermodynamics of monolayers formed by mixtures of phosphatidylcholine/phosphatidylserine. Colloid Surface B.

[35] Karwowska K., Skrodzka E., Kotyńska J., Petelska A.D. (2020). Equilibria in DPPC-Diosgenin and DPPC-Diosgenin Acetate Bilayer Lipid Membranes: Interfacial Tension and Microelectrophoretic Studies. Coatings.

[36] Petelska A.D., Kotyńska J., Figaszewski Z.A. (2015). The effect of fatal carbon monoxide poisoning on the equilibria between cell membranes and the electrolyte solution. J. Membrane Biol..

[37] Petelska A.D., Naumowicz M., Figaszewski Z.A. (2006). The interfacial tension of the lipid membrane formed from lipid-cholesterol and lipid-lipid systems. Cell Biochem. Biophys..

[38] Petelska A.D., Figaszewski Z.A. (2000). Effect of pH on the interfacial tension of lipid bilayer membrane. Biophys. J..

[39] (1974). Engineers Handbook.

[40] Petelska A.D., Figaszewski Z.A. (2003). Acid-base equilibria at interface separating electrolyte solution and lipid bilayer formed from phosphatidylcholine. Biophys. Chem..

[41] Gruen D.W.R., Wolfe J. (1982). Lateral tensions and pressures in membranes and lipid monolayers. Biochim. Biophys. Acta.

[42] Petelska A.D., Figaszewski Z.A. (2011). Interfacial Tension of the Lipid Membrane Formed from Phosphatidylcholine –Decanoic Acid and Phosphatidylcholine–Decylamine Systems. J. Membrane Biol..

[43] Petelska A.D., Figaszewski Z.A. (2011). The equilibria of phosphatidylcholine-fatty acid and phosphatidylcholine-amine in monolayers at the air/water interface. Colloids Surface B.

[44] Singleton W.S., Gray M.S., Brown M.L., White J.L. (1965). Chromatographically homogenous lecithin from egg phospholipids. J. Am. Oil Chem. Soc..

